# Associations of resting plasma adenosine triphosphate, antioxidant capacity, and oxidative stress biomarkers with cardiorespiratory fitness in combat sport athletes: a pilot cross-sectional study

**DOI:** 10.3389/fphys.2026.1863045

**Published:** 2026-08-12

**Authors:** Jinling Huang, Lüfeng Miao, Yangjun Liu

**Affiliations:** 1 Department of Basic Teaching, Pujiang College, Nanjing University of Technology, Nanjing, China; 2 School of Sports Training, Nanjing Sport Institute, Nanjing, China; 3 School of Physical Education and Health, Chengdu University of Traditional Chinese Medicine, Chengdu, China

**Keywords:** combat sport athletes, Cardiorespiratory fitness, VO_2_max, Total antioxidant capacity, Malondialdehyde

## Abstract

**Background and study aim:**

Combat sports involve repeated high-intensity efforts separated by short recovery periods, creating substantial demands on aerobic function and recovery. However, little is known about whether resting metabolic and redox-related biomarkers are associated with cardiorespiratory fitness in combat sport athletes. This pilot cross-sectional study examined the associations of resting plasma adenosine triphosphate (ATP), total antioxidant capacity (T-AOC), superoxide dismutase (SOD), malondialdehyde (MDA), and glutathione peroxidase (GPX) with maximal oxygen uptake (VO_2_max) and relative VO_2_max.

**Materials and methods:**

Resting venous blood samples were collected once after at least 8 h of fasting and before a graded treadmill exercise test; no post-exercise samples were obtained. Cardiorespiratory fitness was evaluated using a graded treadmill exercise test, with VO_2_max, relative VO_2_max, respiratory exchange ratio, exercise heart rate, and exercise duration recorded. Statistical analyses included descriptive statistics, Spearman and partial correlations, multiple linear regression, and decision tree modeling, with additional robustness checks using Bootstrap confidence intervals and permutation tests.

**Results:**

Twenty-one combat sport athletes were included. Mean VO_2_max was 2937.19 ± 643.93 mL/min, and mean relative VO_2_max was 42.38 ± 8.80 mL/kg/min.s. T-AOC was strongly positively correlated with both VO_2_max (Spearman’s ρ = 0.723, p < 0.01) and relative VO_2_max (Spearman’s ρ = 0.678, p < 0.01), and these associations remained significant in partial correlation analyses adjusted for age, sex, height, and body mass. MDA was moderately correlated with relative VO_2_max (ρ = 0.454, p < 0.05). In multivariable models, T-AOC was the only biomarker significantly associated with VO_2_max and, together with MDA, was associated with relative VO_2_max. Partial correlation, decision tree, Bootstrap, and permutation analyses consistently supported T-AOC as the most stable signal across analytical approaches, suggesting that its association with cardiorespiratory fitness was not model-specific. By contrast, ATP, SOD, and GPX showed limited independent explanatory value.

**Conclusions:**

Combat sport athletes in this pilot sample demonstrated high cardiorespiratory fitness. In this pilot sample, resting T-AOC showed the most consistent positive association with VO_2_max and relative VO_2_max, while MDA showed an exploratory association with relative VO_2_max. Because biomarkers were measured once before exercise, these results represent baseline cross-sectional associations and do not establish causality, acute exercise responses, or recovery status. Larger longitudinal studies are needed to confirm these findings.

## 1 Introduction

Cardiorespiratory fitness is commonly assessed using maximal oxygen uptake (VO_2_max) and body-mass-normalized VO_2_max (relative VO_2_max), which are widely used in sports science and practice (Wells and Norris, 2009; Ahmed et al., 2024). VO_2_max directly reflects the body’s ability to uptake, transport, and utilize oxygen under high-intensity exercise, serving as a key determinant of endurance performance and closely linked to cardiovascular health (Lee and Zhang, 2021). Relative VO_2_max, by accounting for body mass, provides a more accurate evaluation of metabolic efficiency across individuals and is particularly meaningful when comparing athletes of different body types (Tongwu et al., 2025). Previous studies have demonstrated that VO_2_max can be significantly improved through regular exercise training in the general population, highlighting the plasticity of aerobic fitness (Butcher et al., 2015; Mohajan and Mohajan, 2023). For athletes, high cardiorespiratory fitness not only underpins the capacity to withstand intense training and competition but also serves as a physiological foundation for optimal recovery, delayed fatigue, and enhanced performance (Furrer et al., 2023).

Combat sports, as an important component of the Olympic program, include disciplines such as judo, taekwondo, boxing, and Brazilian jiu-jitsu (Barley and Harms, 2021; Dou, 2025). They are generally characterized by high-intensity intermittent loading, requiring athletes to perform explosive actions within a very short period while also recovering rapidly between bouts to maintain sustained combat readiness (Guard, 2025). This “high-intensity intermittent–recovery” cycle places substantial demands on both aerobic and anaerobic energy systems. Cross-sectional studies have reported that combat sport athletes typically exhibit higher VO_2_max values than the general population, indicating a strong baseline of cardiorespiratory function (Carayanni et al., 2022; Gwotmut, 2023). However, some athletes within these sports still present with elevated body fat percentages, insufficient muscular strength, or uneven distribution of cardiorespiratory efficiency (Bueno et al., 2022). These observations suggest that beyond traditional physical fitness factors, variations in energy metabolism and antioxidant defense systems may profoundly influence adaptation to training and competition in combat sports. Nevertheless, most existing research has focused on general populations or team sport athletes, leaving systematic data on combat sport athletes relatively scarce (Franchini, 2023).

From a physiological perspective, high-intensity exercise is often accompanied by excessive production of reactive oxygen species (ROS), leading to elevated oxidative stress and subsequent cellular damage (Flensted-Jensen et al., 2021). The body relies on antioxidant systems such as SOD, GPX, and total antioxidant capacity (T-AOC) to neutralize free radicals and maintain cellular homeostasis (Meng and Su, 2024). MDA, a product of lipid peroxidation, is commonly used as a sensitive marker of oxidative damage, with elevated levels indicating accumulated oxidative stress (Mohideen et al., 2023). During high-intensity exercise, increased production of reactive oxygen species (ROS) may alter redox balance (Flensted-Jensen et al., 2021). Antioxidant defenses include superoxide dismutase (SOD), glutathione peroxidase (GPX), and total antioxidant capacity (T-AOC) (Meng and Su, 2024). Malondialdehyde (MDA), a product of lipid peroxidation, is commonly used as a marker of oxidative damage (Mohideen et al., 2023). Adenosine triphosphate (ATP) is central to energy metabolism and also contributes to vascular signaling during exercise (Cardoso et al., 2021; Egan and Sharples, 2023). Prior research indicates that individuals with higher VO_2_max often exhibit stronger antioxidant defenses and lower lipid peroxidation levels, suggesting a positive coupling between cardiorespiratory fitness and antioxidant status (Gao et al., 2025). Different biomarkers carry distinct predictive value: T-AOC is regarded as a key integrative marker; MDA is typically negatively or moderately associated with aerobic capacity; and SOD and GPX are highly responsive to training and nutritional interventions, with GPX changes in particular being closely associated with improvements in VO_2_max in some studies. These findings provide biological underpinnings for understanding cardiorespiratory fitness at the molecular level.

Although research on the relationship between oxidative stress and cardiorespiratory fitness has grown internationally, empirical data specific to combat sport athletes remain insufficient. The training and competition demands of combat sports—intense, intermittent, and combative—may impose unique requirements on energy supply and redox regulation compared with endurance or team-sport athletes. Current evidence on plasma energy metabolism and antioxidant status in combat sport athletes is fragmented, lacking systematic studies to elucidate their relationship with cardiorespiratory fitness.

Given the limited evidence in combat sport athletes, a baseline resting cross-sectional design is an appropriate first step for determining whether interindividual differences in metabolic and redox-related biomarkers are associated with cardiorespiratory fitness under standardized, non-exercise conditions. Collecting blood before the graded exercise test separates resting biomarker status from acute exercise-induced changes and identifies candidate associations for later longitudinal or repeated-sampling studies.

Accordingly, this pilot study examined the associations of resting plasma adenosine triphosphate (ATP), total antioxidant capacity (T-AOC), superoxide dismutase (SOD), malondialdehyde (MDA), and glutathione peroxidase (GPX) with maximal oxygen uptake (VO_2_max) and relative VO_2_max in combat sport athletes. We hypothesized that T-AOC would be positively associated with both fitness outcomes; associations involving the other biomarkers were treated as exploratory.

## 2 Materials and methods

### 2.1 Study design

**Figure 1:**
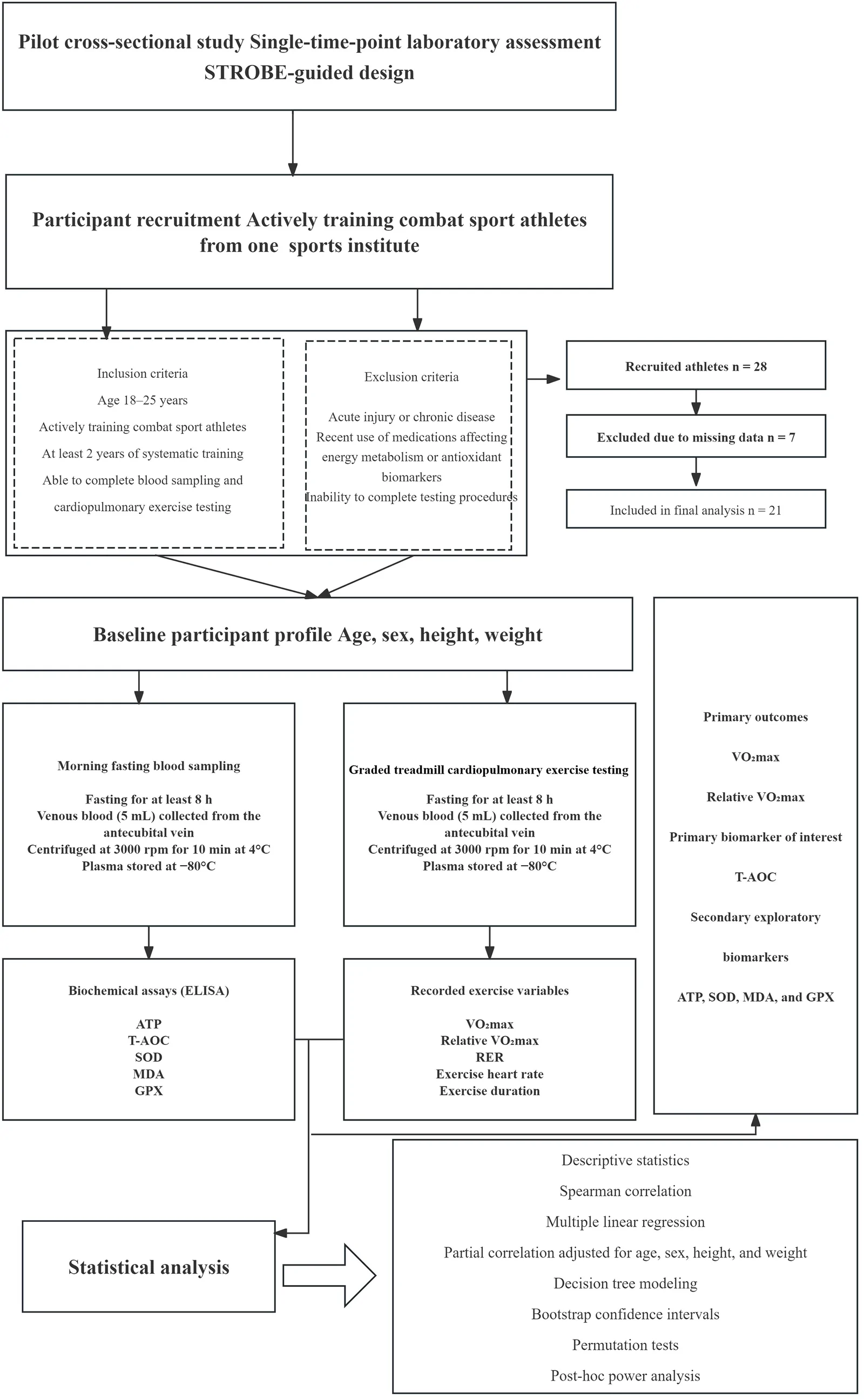
Study design and workflow diagram. Blood sampling was performed only once under resting fasting conditions before exercise testing; no post-exercise biochemical assessment was conducted. SE, standard error; CI, confidence interval; LL, lower limit; UL, upper limit; IQR, interquartile range; Kurt, kurtosis; Skew, skewness; CV, coefficient of variation.VO_2_max, maximal oxygen uptake; Rel VO_2_max, relative oxygen uptake; Ht, height; BM, body mass; RER, respiratory exchange ratio; HR, heart rate; Ex Dur, exercise duration; ATP, adenosine triphosphate; T-AOC, total antioxidant capacity; SOD, superoxide dismutase; MDA, malondialdehyde; GPX, glutathione peroxidase.

This study followed the STROBE checklist for cross-sectional studies and adopted a cross-sectional design to investigate the relationship between plasma energy metabolism, antioxidant biomarkers, and cardiorespiratory fitness (**Figure 1**). All assessments were conducted at a single time point to avoid the influence of interventions or follow-ups on the results, thereby providing a more objective reflection of individual differences under specific physiological conditions. Resting plasma biomarkers were collected before cardiopulmonary exercise testing, and cardiorespiratory parameters were subsequently obtained during the graded exercise test, integrating perspectives from exercise physiology and molecular biology to establish an interdisciplinary analytical framework. All participants successfully completed both cardiopulmonary exercise testing and venous blood sampling, ensuring the comparability and completeness of the data. The design was exploratory in nature, consistent with the positioning of a pilot study, and aimed to provide both scientific evidence and practical reference for elucidating the relationship between antioxidant capacity and cardiorespiratory fitness.

### 2.2 Participants

Participants were recruited by convenience sampling from combat sport teams at ** Sports Institute, including athletes from [boxing, taekwondo, judo, wrestling, and wushu sanda]. A total of 28 athletes were initially recruited; after excluding seven athletes with missing data, 21 athletes were included in the final analysis. The final sample consisted of 16 males and 5 females, all of whom were student-athletes with systematic training backgrounds and relatively uniform training conditions. Prior to the formal experiment, participants were confirmed to be in good health, with no history of major diseases or acute injuries, and capable of completing cardiopulmonary exercise testing and blood sampling. All measurements were conducted under identical laboratory conditions to minimize external interference. The core of the study was to simultaneously collect cardiopulmonary function data and plasma energy metabolism and antioxidant indicators, thereby exploring their associations and providing empirical evidence for evaluating athletes’ cardiorespiratory fitness. The cross-sectional design allowed for capturing interindividual differences at a single time point, making it suitable for exploring potential correlations rather than causal inferences. Despite the limited sample size, this design provided preliminary evidence for subsequent longitudinal follow-ups or interventional studies and laid a foundation for further investigation of the relationship between energy metabolism characteristics and cardiorespiratory fitness in combat sport athletes.

### 2.3 Inclusion and exclusion criteria

All participants were student-athletes undergoing regular training, with continuous and systematic sport-specific training experience, representing the typical physical and functional profiles of combat sport athletes. Strict control of participant characteristics was implemented to ensure sample homogeneity and reliability of results. Each athlete underwent baseline information collection and health assessments before the experiment to confirm eligibility. Unified laboratory settings and standardized procedures were applied to minimize external interference and establish a solid basis for subsequent analysis. Inclusion criteria: Actively training combat sport athletes aged 18–25 years, regardless of sex, with at least two years of systematic training.

*Exclusion criteria:* Presence of acute injuries, chronic diseases, or a recent history of major diseases or surgeries before the experiment; recent use of medications potentially affecting energy metabolism or antioxidant biomarkers; inability to complete cardiopulmonary exercise testing or blood sampling.

Applying these criteria ensured consistency in health status and training conditions, allowing the findings to more accurately reflect the physiological and functional characteristics of combat sport athletes.

### 2.4 Data collection and measurements

#### Demographic and anthropometric indicators

To ensure completeness and comparability of participant characteristics, basic demographic and anthropometric data were collected before the formal experiment. Variables included age, sex, height, and body mass. Age and sex were recorded by self-report. Height was measured using a standard stadiometer with participants standing barefoot, accurate to 0.1 cm. Body mass was measured using an electronic scale, with participants wearing light clothing and under fasting conditions, accurate to 0.1 kg. All measurements were performed by the same trained research team during the same time window to minimize human and environmental variability. These indicators were used both to describe the sample characteristics and to serve as potential covariates in statistical analyses.

### 2.5 Assessment of cardiorespiratory fitness

Cardiorespiratory fitness was evaluated using a graded exercise test (GXT) on a treadmill, with volitional exhaustion as the termination criterion. Participants wore a portable metabolic analyzer (COSMED K5), and expired gases and ventilation were continuously measured through a face mask to calculate oxygen uptake and determine VO_2_max, the gold standard for assessing cardiorespiratory fitness. Participants were instructed to maintain a normal diet and rest, and to avoid strenuous exercise and caffeine intake prior to testing to reduce external interference. The exercise protocol followed a progressive incremental model, starting at low speed or incline, with stepwise increases in intensity until exhaustion or meeting termination criteria. VO_2_max was defined based on criteria including: oxygen uptake reaching a plateau despite increased workload, respiratory exchange ratio (RER) ≥ 1.10, heart rate reaching or exceeding 90% of age-predicted maximum, and voluntary exhaustion. Additional parameters recorded included RER, exercise heart rate, and exercise duration. Relative oxygen uptake (ml/kg/min) was also calculated to comprehensively reflect aerobic capacity and endurance. All tests were supervised by certified exercise testing professionals and performed according to exercise physiology standards to ensure both participant safety and data reliability.

### 2.6 Plasma energy metabolism and antioxidant biomarkers

Resting plasma biomarkers were measured after at least 8 h of fasting and within a standardized time window to reduce variation related to recent food intake and time of day to minimize the influence of diet and circadian rhythms. Participants fasted for at least 8 hours prior to testing. Venous blood (5 ml) was drawn from the antecubital vein by trained personnel under resting conditions. Samples were immediately placed in anticoagulant tubes, centrifuged at 3000 rpm for 10 minutes at 4 °C, and plasma was transferred into pre-cooled tubes, stored at –80 °C until analysis. Enzyme-linked immunosorbent assays (ELISA) were performed using commercial kits from the same batch, strictly following manufacturer protocols. Biomarkers included: Biomarkers included adenosine triphosphate (ATP) concentration, total antioxidant capacity (T-AOC), superoxide dismutase (SOD) activity, malondialdehyde (MDA) concentration, and glutathione peroxidase (GPX) activity. To ensure reliability and comparability, all experiments were conducted by the same trained laboratory staff under identical conditions, with blinded sample coding to avoid subjective bias. The entire procedure followed strict quality control standards to minimize inter-assay variability and random error, ensuring scientific accuracy of the results. All blood samples were collected before the exercise test; no post-exercise biochemical assessment was performed. Therefore, biochemical outcomes represent resting baseline status only.

### 2.7 Sampling procedure

All sample collection and analyses were conducted between May 20 and June 10, 2025. To ensure systematic and consistent procedures, all experimental stages were scheduled within standardized time windows and carried out by a fixed technical team. The procedure included participant preparation, data and sample collection, standardized experimental conditions, and centralized laboratory analyses. All measurements were conducted on the same platform, using identical equipment and reagents from the same batch to minimize inter-assay variability. Sample coding, storage, and analysis were tracked throughout to ensure information integrity at each stage.

### 2.8 Statistical analysis

All data were processed and analyzed using R software to explore the relationships between plasma energy metabolism, antioxidant biomarkers, and cardiorespiratory fitness in combat sport athletes. VO_2_max and relative VO_2_max were treated as the primary outcomes. Based on the study hypothesis, T-AOC was considered the primary biomarker of interest, whereas ATP, SOD, MDA, and GPX were analyzed as secondary exploratory biomarkers. Descriptive statistics were used to present the basic characteristics of each variable, expressed as mean ± standard deviation (Mean ± SD), with additional calculations of 95% confidence intervals (95% CI), interquartile range (IQR), skewness, kurtosis, and coefficient of variation (CV) to characterize sample distribution and individual variability. Correlation analyses were conducted according to data distribution; when normality assumptions were not met, Spearman’s rank correlation test was applied to evaluate associations between plasma biomarkers and cardiorespiratory fitness parameters, thereby identifying potential univariate trends. Spearman’s rank correlation coefficients were reported as ρ. Partial correlation coefficients were reported as r_p_. R^2^ and adjusted R^2^ were reported only as coefficients of determination for regression models, not as correlation coefficients. To further assess the combined effects of multiple factors, multiple linear regression models were developed with VO_2_max and relative VO_2_max (ml/kg/min) as dependent variables and energy metabolism and antioxidant biomarkers as independent variables. Associations were evaluated using standardized regression coefficients (Beta), significance levels (p-values), and coefficients of determination (R^2^ and adjusted R^2^), while variance inflation factor (VIF) and tolerance were applied to detect multicollinearity and ensure model robustness.

Partial correlation analyses were additionally performed to re-examine associations between biochemical indicators and cardiorespiratory fitness after controlling for age, sex, height, and body mass. This adjustment was used to reduce potential confounding from demographic and anthropometric variables. Considering the possibility of complex interactions among variables, Exploratory regression-tree models were fitted separately for VO_2_max and relative VO_2_max to describe variable-importance patterns within the sample, identifying key branching variables and thresholds to visualize the role of energy metabolism and antioxidant markers in distinguishing performance. Given the limited sample size, Bootstrap confidence intervals and permutation tests were incorporated to validate the robustness of correlation and regression findings and to reduce small-sample bias. Finally, *post-hoc* power analyses were conducted, calculating statistical power at different correlation coefficients (r values) to assess the sample’s detection ability for strong and moderate effects, thereby clarifying the explanatory strength and limitations of the study’s conclusions. All statistical tests were two-tailed, with significance set at p < 0.05.

### 2.9 Ethics and data management

This study was approved by the Ethics Committee of *** Institute (Approval No. RT-2025-12) and conducted in strict accordance with the principles of the Declaration of Helsinki. Prior to data collection, all participants were provided with detailed information regarding the study’s purpose, procedures, and potential risks, and written informed consent was obtained to ensure voluntary participation and compliance. All data collected during the study were anonymized and used solely for scientific research purposes, accessible only to core members of the research team to guarantee data security and confidentiality.

## 3 Results

### 3.1 General characteristics of participants

A total of 28 combat sport athletes were recruited by convenience sampling from [boxing, taekwondo, judo, wrestling, and wushu sanda] teams; due to missing data, 21 athletes were ultimately included in the analysis. The final sample consisted of 16 males and 5 females. The average height was 173.21 ± 9.32 cm and body mass was 70.88 ± 16.63 kg. Regarding cardiorespiratory fitness, the mean VO_2_max was 2937.19 ± 643.93 ml/min, relative oxygen uptake was 42.38 ± 8.80 ml/kg/min, respiratory exchange ratio was 1.21 ± 0.05, exercise heart rate reached 195.62 ± 8.41 bpm, and exercise duration was approximately 574.71 ± 98.63 seconds, all reflecting a high level of tolerance to exercise load. Plasma metabolic and antioxidant indicators showed an ATP concentration of 2.02 ± 0.77 μmol/ml, total antioxidant capacity of 1.29 ± 0.19 μmol/ml, SOD activity of 4.83 ± 0.82 U/ml, MDA concentration of 0.98 ± 0.28 nmol/ml, and GPX activity of 141.45 ± 29.62 U/ml. Coefficients of variation ranged from 4.1% to 38.1%, with greater variability observed in ATP and MDA, indicating pronounced individual differences, whereas respiratory exchange ratio and heart rate demonstrated low variability and concentrated distributions. Skewness and kurtosis analyses suggested that most variables approximated normal distributions, with the exception of ATP and MDA, which displayed skewed distributions. Overall, participants were relatively homogeneous in demographic and cardiorespiratory measures, while antioxidant and energy metabolism markers exhibited heterogeneity, reflecting individual differences in energy supply and oxidative stress levels (**Table 1**).

**Table 1:** Descriptive statistics of demographic characteristics, cardiorespiratory fitness parameters, and plasma biomarkers in combat sport athletes.

Name	Mean ± SD	SE	95% CI (LL)	95% CI (UL)	IQR	Kurt	Skew	CV
Age	20.238 ± 0.831	0.181	19.883	20.593	1	0.417	0.66	4.11%
Sex	1.238 ± 0.436	0.095	1.051	1.425	0.5	-0.276	1.327	35.25%
Ht	173.214 ± 9.315	2.033	169.23	177.198	14	-0.471	0.163	5.38%
Wt	70.881 ± 16.627	3.628	63.77	77.992	17.85	1.597	1.243	23.46%
VO_2_max	2937.190 ± 643.929	140.517	2661.782	3212.599	844	0.24	0.428	21.92%
RVO_2_max	42.381 ± 8.795	1.919	38.619	46.142	12.06	-0.059	-0.107	20.75%
RQ	1.208 ± 0.054	0.012	1.185	1.231	0.065	0.612	-0.724	4.44%
HR	195.619 ± 8.405	1.834	192.024	199.214	14.5	-0.799	-0.098	4.30%
DurEx	574.714 ± 98.628	21.522	532.531	616.897	172.5	-1.199	-0.318	17.16%
ATP	2.020 ± 0.770	0.168	1.691	2.349	0.695	2.525	1.125	38.10%
TAC	1.294 ± 0.185	0.04	1.215	1.373	0.33	-0.148	0.37	14.29%
SOD	4.828 ± 0.822	0.179	4.476	5.18	1.405	-0.664	-0.137	17.03%
MDA	0.982 ± 0.283	0.062	0.861	1.104	0.3	1.725	-0.23	28.85%
GPX	141.450 ± 29.619	6.463	128.782	154.117	58.355	-1.039	0.365	20.94%

### 3.2 Correlations between cardiorespiratory fitness and plasma biomarkers

**Figure 2:**
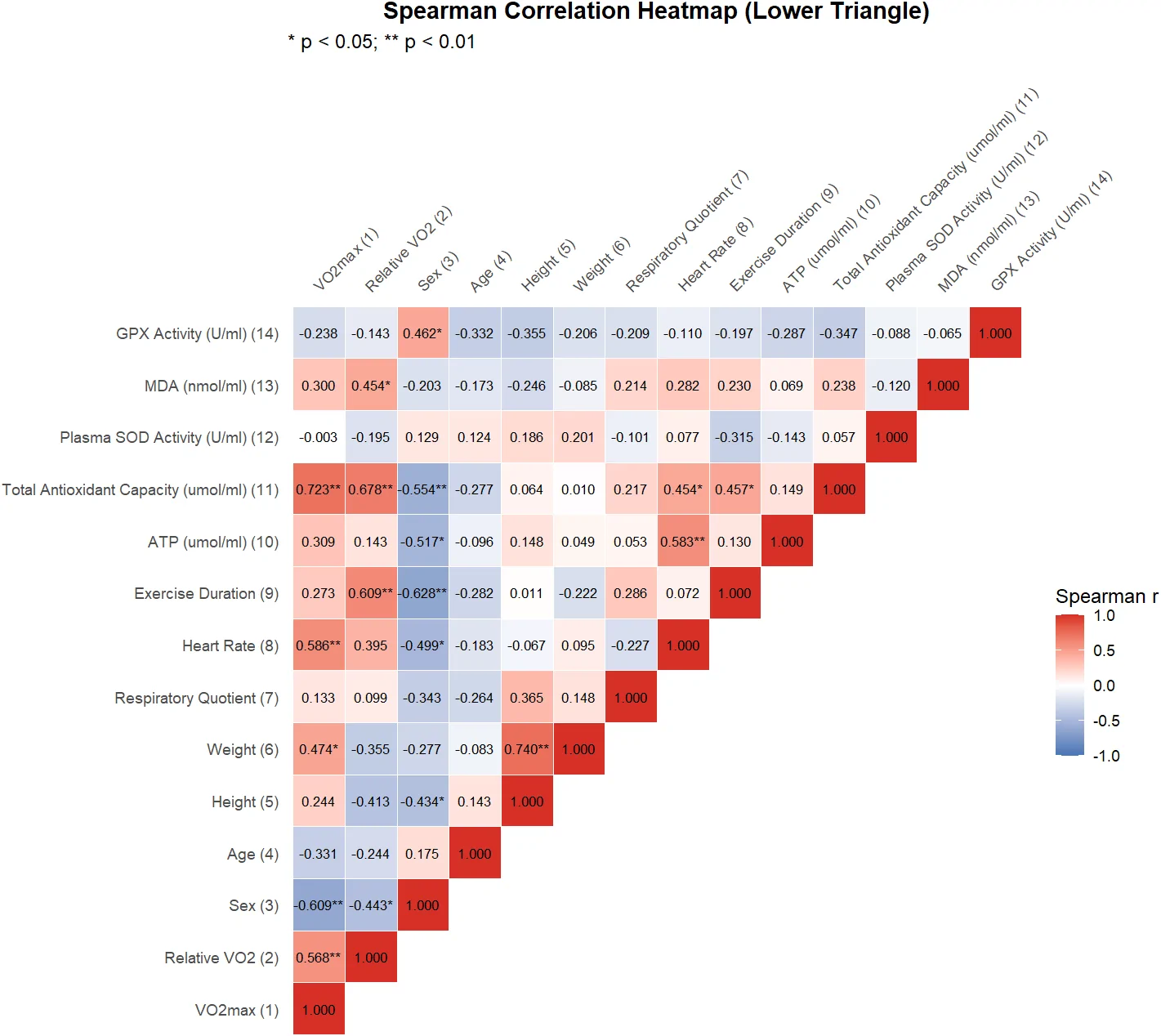
Heatmap of correlation coefficients between plasma biomarkers and cardiorespiratory fitness parameters.

Correlation analyses (**Table 2**; **Figure 2**) indicated that sex was negatively associated with VO_2_max (ρ= –0.609, p < 0.01) and relative oxygen uptake (ρ= –0.443, p < 0.05), suggesting lower oxygen uptake in females. body mass correlated positively with VO_2_max (ρ= 0.474, p < 0.05), while height correlated negatively with relative oxygen uptake (ρ= –0.413, p < 0.05), indicating that body size influences absolute and relative oxygen uptake differently. Heart rate was significantly correlated with VO_2_max (ρ= 0.586, p < 0.01), and exercise duration was strongly associated with relative oxygen uptake (ρ= 0.609, p < 0.01), underscoring the close relationship between exercise tolerance and aerobic capacity. Among biochemical markers, total antioxidant capacity was strongly correlated with both VO_2_max (ρ= 0.723, p < 0.01) and relative oxygen uptake (ρ= 0.678, p < 0.01), emerging as the most prominent finding. MDA showed a moderate correlation with relative oxygen uptake (ρ= 0.454, p < 0.05), linking oxidative stress products to aerobic capacity. In contrast, ATP, SOD, and GPX showed no significant associations with cardiorespiratory fitness. These results suggest that total antioxidant capacity was the strongest resting biomarker associated with cardiorespiratory fitness. in combat sport athletes, while the role of MDA warrants further investigation.

**Table 2:** Correlation analysis between plasma biomarkers and cardiorespiratory fitness parameters in combat sport athletes.

Variable	1	2	3	4	5	6	7	8	9	10	11	12	13	14
VO_2_max (Wells and Norris, 2009)	1													
Rel VO_2_max (Ahmed et al., 2024)	0.568**	1												
Sex (Lee and Zhang, 2021)	-0.609**	-0.443*	1											
Age (Tongwu et al., 2025)	-0.331	-0.244	0.175	1										
Ht (cm) (Mohajan and Mohajan, 2023)	0.244	-0.413	-0.434*	0.143	1									
Wt (kg) (Butcher et al., 2015)	0.474*	-0.355	-0.277	-0.083	0.740**	1								
RER (Furrer et al., 2023)	0.133	0.099	-0.343	-0.264	0.365	0.148	1							
HR (bpm) (Barley and Harms, 2021)	0.586**	0.395	-0.499*	-0.183	-0.067	0.095	-0.227	1						
Ex Dur (s) (Dou, 2025)	0.273	0.609**	-0.628**	-0.282	0.011	-0.222	0.286	0.072	1					
ATP (µmol/ml) (Guard, 2025)	0.309	0.143	-0.517*	-0.096	0.148	0.049	0.053	0.583**	0.13	1				
T-AOC (µmol/ml) (Carayanni et al., 2022)	0.723**	0.678**	-0.554**	-0.277	0.064	0.01	0.217	0.454*	0.457*	0.149	1			
SOD (U/ml) (Gwotmut, 2023)	-0.003	-0.195	0.129	0.124	0.186	0.201	-0.101	0.077	-0.315	-0.143	0.057	1		
MDA (nmol/ml) (Bueno et al., 2022)	0.3	0.454*	-0.203	-0.173	-0.246	-0.085	0.214	0.282	0.23	0.069	0.238	-0.12	1	
GPX (U/ml) (Franchini, 2023)	-0.238	-0.143	0.462*	-0.332	-0.355	-0.206	-0.209	-0.11	-0.197	-0.287	-0.347	-0.088	-0.065	1

Values are Spearman’s rank correlation coefficients (ρ).

* p<0.05 ** p<0.01;. VO_2_max, maximal oxygen uptake; Rel VO_2_max, relative oxygen uptake; Ht, height; Wt, weight; RER, respiratory exchange ratio; HR, heart rate; Ex Dur, exercise duration; ATP, adenosine triphosphate; T-AOC, total antioxidant capacity; SOD, superoxide dismutase; MDA, malondialdehyde; GPX, glutathione peroxidase.

### 3.3 Linear regression analysis of VO_2_max

**Figure 3:**
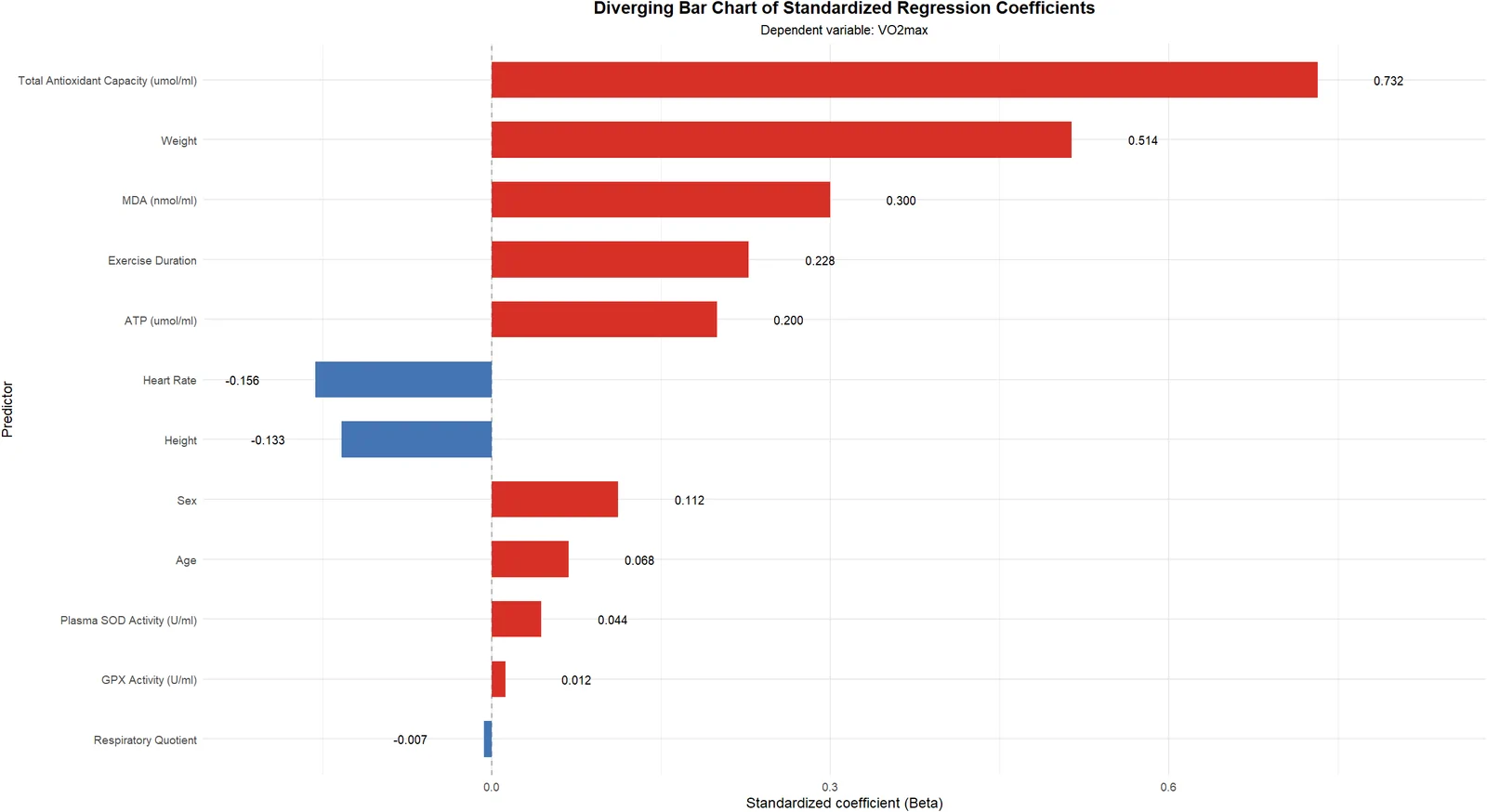
Bar chart of standardized regression coefficients for predictors of maximal oxygen uptake (VO_2_max).

Multivariate regression analyses with VO_2_max as the dependent variable (**Table 3**) identified total antioxidant capacity as the only biomarker significantly associated with VO_2_max (B = 2550.653, p = 0.023), with a standardized coefficient of 0.732, indicating a strong positive association between antioxidant capacity and VO_2_max. Other variables, including age, sex, height, weight, respiratory exchange ratio, heart rate, exercise duration, and biomarkers (ATP, SOD, MDA, GPX), did not demonstrate independent predictive value (**Figure 3**). The model yielded R^2^ = 0.830 and adjusted R^2^ = 0.575. The overall model narrowly missed the conventional significance threshold (F = 3.253, p = 0.051), and elevated variance inflation factors for sex, height, and body mass indicated multicollinearity. Given the small sample and the number of model terms, the positive T-AOC coefficient should be interpreted as exploratory.

**Table 3:** Multiple linear regression analysis of VO_2_max with demographic, physiological, and plasma biomarker predictors.

Predictor	Unstandardized coefficients		Standardized coefficients	t	p	Collinearity diagnostics	
	B	Standard error	Beta			VIF	Tolerance
Constant	-1072.263	9021.735	-	-0.119	0.908	-	-
Age	52.716	174.698	0.068	0.302	0.771	2.39	0.418
Sex	165.877	660.468	0.112	0.251	0.808	9.426	0.106
Height	-9.172	27.67	-0.133	-0.331	0.749	7.536	0.133
Weight	19.898	16.408	0.514	1.213	0.26	8.443	0.118
Respiratory Quotient	-88.425	2867.806	-0.007	-0.031	0.976	2.683	0.373
Heart Rate	-11.978	25.397	-0.156	-0.472	0.65	5.169	0.193
Duration of Continuous Exercise	1.489	2.572	0.228	0.579	0.579	7.302	0.137
ATP Content (µmol/ml)	167.43	237.345	0.2	0.705	0.501	3.786	0.264
Total Antioxidant Capacity (µmol/ml)	2550.653	908.947	0.732	2.806	0.023*	3.205	0.312
Plasma SOD Activity (U/ml)	34.28	136.373	0.044	0.251	0.808	1.426	0.701
MDA Content (nmol/ml)	680.907	428.688	0.3	1.588	0.151	1.675	0.597
GPX Activity (U/ml)	0.255	4.865	0.012	0.052	0.959	2.355	0.425
R^2^	0.83						
Adjusted R^2^	0.575						
F	F (12,8)=3.253,p=0.051						
D–W Value	1.89						

Dependent Variable = Maximal Oxygen Uptake.

*p<0.05 **p<0.01.

### 3.4 Linear regression analysis of relative VO_2_max

**Figure 4:**
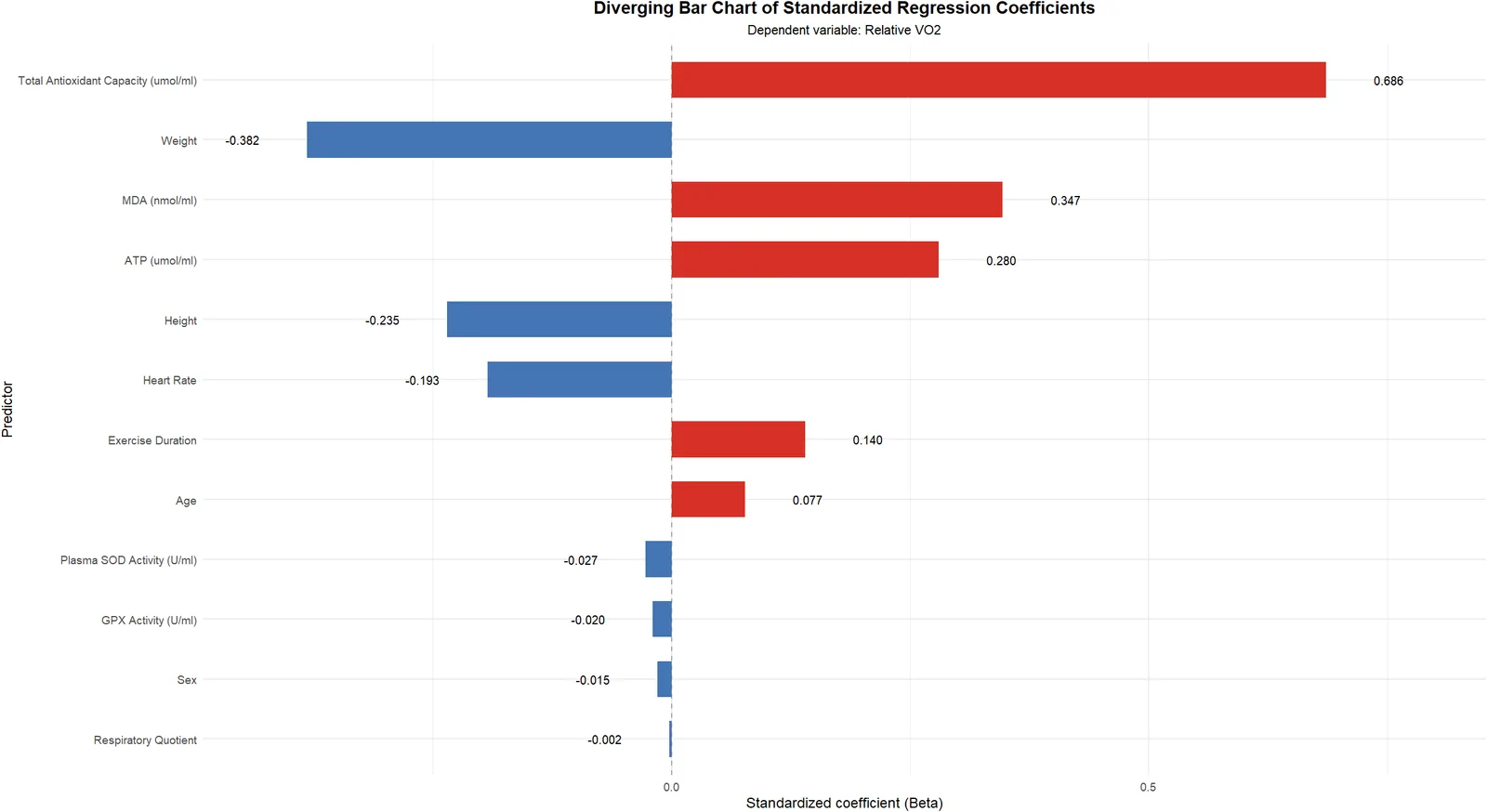
Bar chart of standardized regression coefficients for predictors of relative maximal oxygen uptake (relative VO_2_max).

When relative VO_2_max was used as the dependent variable (**Table 4**), within the fitted model, T-AOC and MDA were positively associated with relative VO_2_max. Total antioxidant capacity had a regression coefficient of 32.64 (p = 0.009) and a standardized coefficient of 0.686, indicating a strong positive association and confirming its stability and prominence within the model. MDA concentration showed a regression coefficient of 10.77 (p = 0.045) and a standardized coefficient of 0.347, also positively associated with relative oxygen uptake, suggesting a role for oxidative stress products in aerobic capacity (**Figure 4**). Other variables were not significant. The overall model fit was strong, with R^2^ = 0.898 and adjusted R^2^ = 0.746, indicating that the primary predictors explained approximately 75% of the variance in relative VO_2_max. The model was statistically significant (F = 5.887, p = 0.009), with acceptable residual independence (D-W =1.755), confirming robustness. Although other biochemical markers did not reach significance, their directional trends suggested possible indirect effects or limitations related to the sample size. Collectively, these results underscore the association between antioxidant capacity and cardiorespiratory fitness among athletes and highlight the potential value of MDA in explaining individual differences in aerobic performance.

**Table 4:** Multiple linear regression analysis of relative VO_2_max with demographic, physiological, and plasma biomarker predictors.

Predictor	Unstandardized coefficients		Standardized coefficients	t	p	Collinearity diagnostics	
	B	Standard error	Beta			VIF	Tolerance
Constant	54.797	95.294	–	0.575	0.581	–	–
Age	0.81	1.845	0.077	0.439	0.672	2.39	0.418
Sex	-0.305	6.976	-0.015	-0.044	0.966	9.426	0.106
Height	-0.222	0.292	-0.235	-0.76	0.469	7.536	0.133
Weight	-0.202	0.173	-0.382	-1.166	0.277	8.443	0.118
Respiratory Quotient	-0.367	30.292	-0.002	-0.012	0.991	2.683	0.373
Heart Rate	-0.201	0.268	-0.193	-0.751	0.474	5.169	0.193
Duration of Continuous Exercise	0.013	0.027	0.14	0.461	0.657	7.302	0.137
ATP Content (µmol/ml)	3.197	2.507	0.28	1.275	0.238	3.786	0.264
Total Antioxidant Capacity (µmol/ml)	32.635	9.601	0.686	3.399	0.009**	3.205	0.312
Plasma SOD Activity (U/ml)	-0.288	1.44	-0.027	-0.2	0.846	1.426	0.701
MDA Content (nmol/ml)	10.772	4.528	0.347	2.379	0.045*	1.675	0.597
GPX Activity (U/ml)	-0.006	0.051	-0.02	-0.117	0.91	2.355	0.425
R^2^	0.898						
Adjusted R^2^	0.746						
F	F (12,8)=5.887,p=0.009						
D–W Value	1.755						

Dependent Variable = Relative Oxygen Uptake.

*p<0.05 **p<0.01.

### 3.5 Partial correlation analysis controlling for confounders

After controlling for potential confounders such as age, sex, height, and weight, partial correlation analysis further clarified the independent associations between biomarkers and cardiorespiratory fitness. As shown in **Table 5**, total antioxidant capacity remained strongly and significantly correlated with VO_2_max (r_p_ = 0.777, p < 0.01) and relative oxygen uptake (r_p_ = 0.580, p < 0.01), highlighting its central role in explaining individual differences in cardiorespiratory function. MDA concentration was also moderately correlated with VO_2_max (r_p_ = 0.441, p < 0.05) and relative oxygen uptake (r_p_ = 0.529, p < 0.05), suggesting that oxidative stress products may reflect variations in aerobic capacity to a certain extent. Heart rate showed a positive correlation with VO_2_max (r_p_ = 0.436, p < 0.05), while exercise duration was closely associated with relative oxygen uptake (r_p_ = 0.633, p < 0.01), underscoring the strong link between physiological load tolerance and cardiorespiratory fitness. In contrast, ATP, SOD, and GPX showed no significant associations, indicating limited explanatory power under cross-sectional conditions. Overall, these findings emphasize total antioxidant capacity as a key predictor of cardiorespiratory fitness while suggesting the potential value of MDA, thereby providing clearer evidence for the interplay between energy metabolism, oxidative stress, and cardiopulmonary function.

**Table 5:** Partial correlation coefficients (r_p_) between plasma biomarkers and cardiorespiratory fitness after controlling for age, sex, height, and body mass.

Variable	Maximal oxygen uptake	Relative oxygen uptake
Age	-0.265	-0.155
Sex	-0.572**	-0.345
Height	0.319	-0.38
Weight	0.433*	-0.480*
Respiratory Quotient	0.194	0.124
Heart Rate	0.436*	0.325
Duration of Continuous Exercise	0.347	0.633**
ATP Content (µmol/ml)	0.112	0.061
Total Antioxidant Capacity (µmol/ml)	0.777**	0.580**
Plasma SOD Activity (U/ml)	0.062	-0.135
MDA Content (nmol/ml)	0.441*	0.529*
GPX Activity (U/ml)	-0.328	-0.198

*p<0.05 **p<0.01.

### 3.6 Decision tree modelling for predicting cardiorespiratory fitness

**Figure 5:**
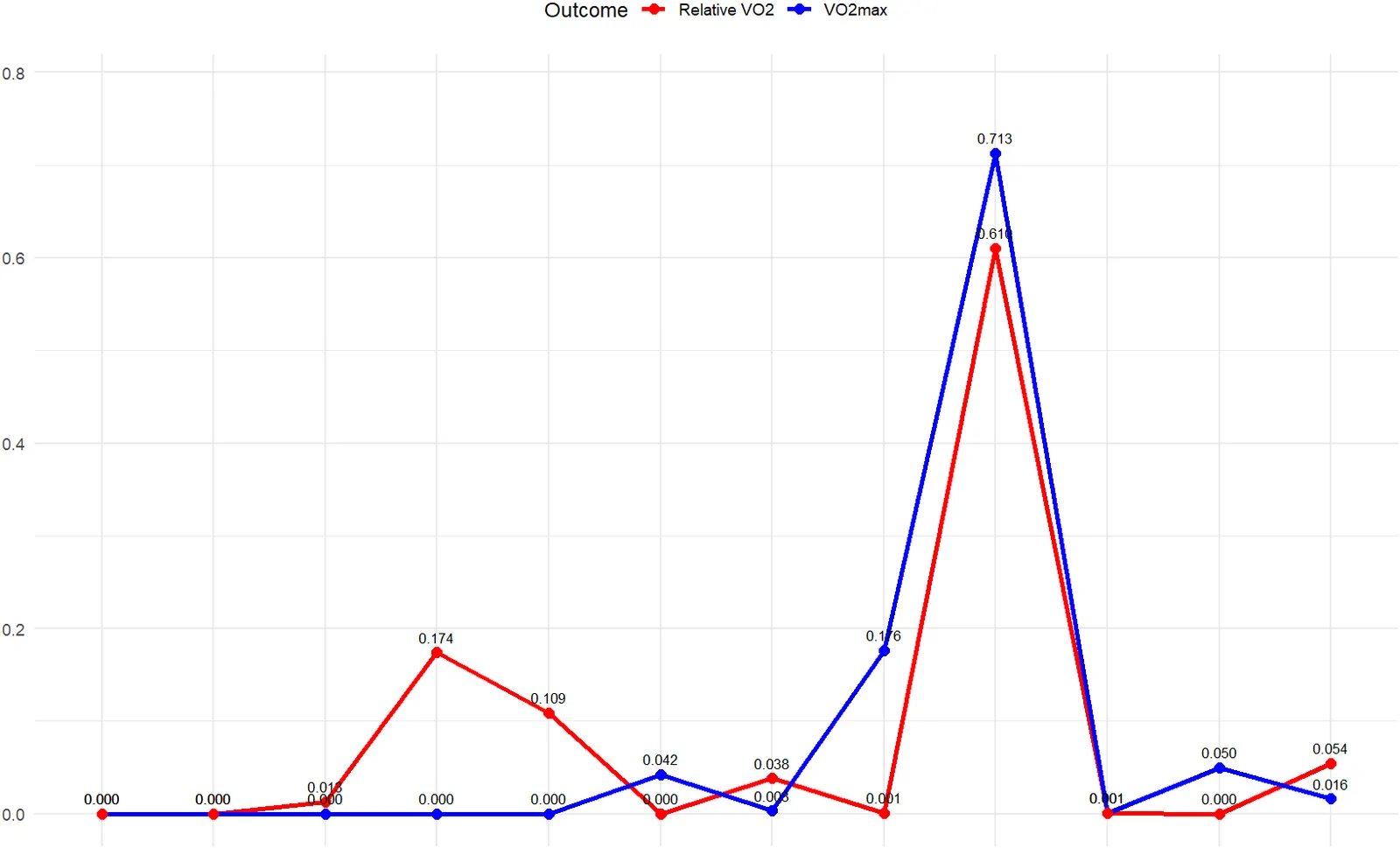
Variable importance weights in decision tree models explaining interindividual variability in VO_2_max and relative VO_2_max.

Decision tree analysis (**Table 6**) revealed that total antioxidant capacity was the most influential factor in differentiating VO_2_max levels, with a weight far exceeding that of other variables (**Figure 5**). ATP concentration and MDA level emerged as secondary nodes, indicating that both energy metabolism and oxidative stress products contribute to aerobic capacity, whereas heart rate and GPX activity had relatively minor roles. For relative VO_2_max, the model again identified total antioxidant capacity as the primary discriminator, followed by body weight and respiratory exchange ratio. This aligns with physiological expectations: body size directly influences relative oxygen consumption, while respiratory exchange ratio partially reflects metabolic efficiency during exercise. In contrast, SOD and GPX carried negligible weight in both models and did not form effective branches, indicating limited independent predictive value. Overall, the decision tree results visually delineated a hierarchical pathway for cardiorespiratory fitness: antioxidant capacity as the primary discriminator, with body size and metabolic indicators providing further stratification. These findings are consistent with correlation and regression analyses, reinforcing the central role of total antioxidant capacity in predicting athletes’ cardiorespiratory function.

**Table 6:** Decision tree analysis for explaining interindividual variability in VO_2_max and relative VO_2_max in combat sport athletes.

Maximal oxygen uptake characteristic weight values		Absolute oxygen uptake characteristic weight values	
Item	Weight value	Item	Weight value
Age	0	Age	0
Sex	0	Sex	0
Height	0	Height	0.013
Weight	0	Weight	0.174
Respiratory Quotient	0	Respiratory Quotient	0.109
Heart Rate	0.042	Heart Rate	0
Duration of Continuous Exercise	0.003	Duration of Continuous Exercise	0.038
ATP Content (µmol/ml)	0.176	ATP Content (µmol/ml)	0.001
Total Antioxidant Capacity (µmol/ml)	0.713	Total Antioxidant Capacity (µmol/ml)	0.61
Plasma SOD Activity (U/ml)	0.001	Plasma SOD Activity (U/ml)	0.001
MDA Content (nmol/ml)	0.05	MDA Content (nmol/ml)	0
GPX Activity (U/ml)	0.016	GPX Activity (U/ml)	0.054

### 3.7 Robustness analysis using bootstrap and permutation tests

**Figure 6:**
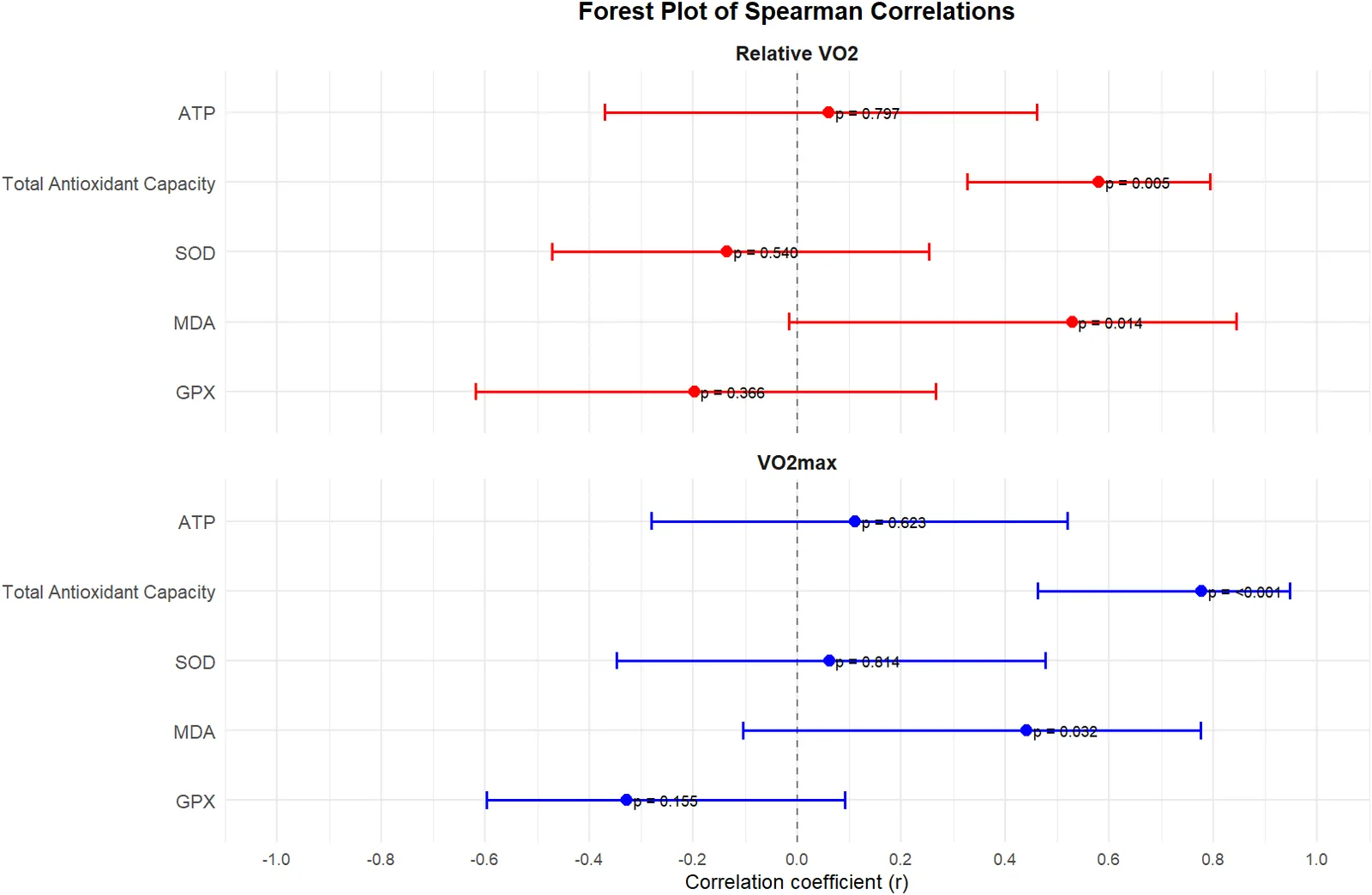
Forest plot of robustness analyses using Bootstrap confidence intervals and permutation tests for VO_2_max and relative VO_2_max.

Robustness checks with Bootstrap and permutation analyses (**Figure 6**) consistently confirmed the stable association between total antioxidant capacity and cardiorespiratory fitness (**Table 7**). Its correlation with VO_2_max was 0.777, with a 95% confidence interval of 0.462–0.948 and p = 0.0001, indicating high statistical significance and reliability. For relative VO_2_max, the correlation was 0.580, with a confidence interval of 0.326–0.794 and p = 0.005, again demonstrating a robust positive association, suggesting that antioxidant capacity independently reflects aerobic capacity across analytical approaches. MDA concentration also showed moderate correlations with both indicators (VO_2_max: r_p_ = 0.441, p = 0.032; relative VO_2_max: r_p_ = 0.529, p = 0.014), though with wider confidence intervals, still pointing to a role of oxidative stress products in explaining variability in cardiorespiratory function. By contrast, ATP, SOD, and GPX showed no significant associations, with correlation coefficients near zero or negative and permutation test p-values > 0.05, indicating limited contributions in a cross-sectional context. The convergence of Bootstrap confidence intervals and permutation results highlights total antioxidant capacity as the most stable correlate, with MDA holding auxiliary value, whereas enzymatic indicators appeared weaker. These findings provide strong statistical support for the relationship between antioxidant status and aerobic capacity in athletes.

**Table 7:** Robustness checks using Bootstrap confidence intervals and permutation tests for associations between plasma biomarkers and cardiorespiratory fitness.

Dependent variable	Independent variable	Correlation coefficient (r)	Bootstrap 95% CI	Permutation test p-value
Maximal Oxygen Uptake	ATP	0.112	[-0.280, 0.520]	0.623
	Total Antioxidant Capacity	0.777	[0.462, 0.948]	0
	SOD	0.062	[-0.347, 0.477]	0.814
	MDA	0.441	[-0.105, 0.776]	0.032
	GPX	-0.328	[-0.598, 0.091]	0.155
Relative Oxygen Uptake	ATP	0.061	[-0.371, 0.461]	0.797
	Total Antioxidant Capacity	0.58	[0.326, 0.794]	0.005
	SOD	-0.135	[-0.472, 0.254]	0.54
	MDA	0.529	[-0.016, 0.844]	0.014
	GPX	-0.198	[-0.619, 0.267]	0.366

### 3.8 *Post-hoc* power analysis

**Figure 7:**
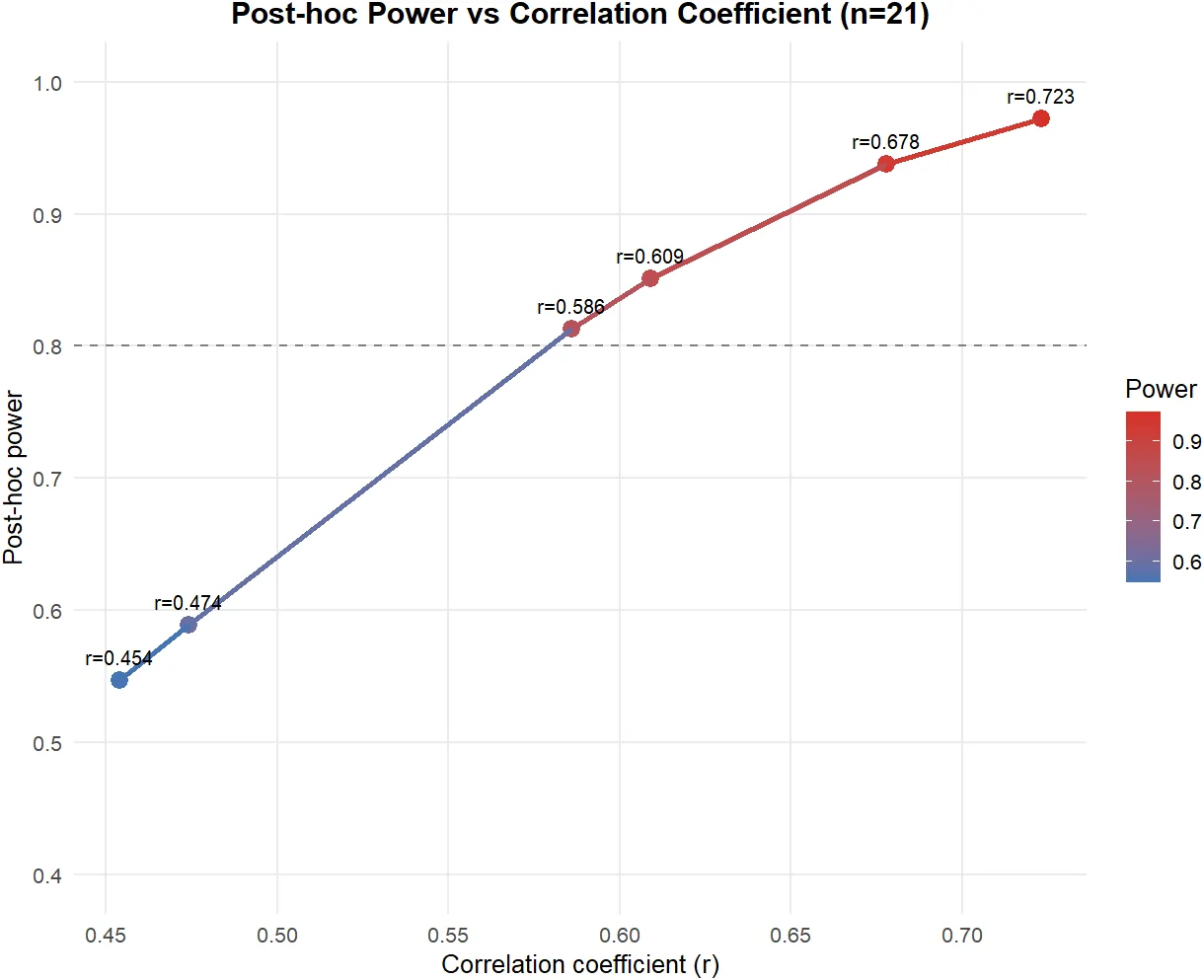
*Post-hoc* statistical power analysis of correlation coefficients.

*Post-hoc* power analysis (**Figure 7**; **Table 8**) demonstrated that with a sample size of 21, strong correlation effects could be reliably detected. For correlation coefficients of 0.723, 0.678, 0.609, and 0.586, the statistical power values were 0.972, 0.938, 0.851, and 0.813, respectively, all exceeding the commonly accepted threshold of 0.80, indicating sufficient sensitivity to capture strong associations. However, for moderate correlations (r_p_ = 0.474 and r_p_ = 0.454), the power values were only 0.589 and 0.547, falling below the ideal level, suggesting that the sample size was insufficient to consistently identify moderate effects. These results indicate that the study’s findings are relatively robust when addressing strong correlations, while observations of moderate correlations remain exploratory and will require larger sample sizes in future research to obtain more conclusive evidence.

**Table 8:** *Post-hoc* statistical power of correlation coefficients in the sample of combat sport athletes.

Correlation coefficient (r)	*Post Hoc* power (n=21)
0.723	0.972
0.678	0.938
0.609	0.851
0.586	0.813
0.474	0.589
0.454	0.547

## 4 Discussion

Before interpreting these findings, it should be emphasized that the present study did not assess acute oxidative stress or antioxidant responses to maximal exercise. Blood biomarkers were measured only once under standardized resting fasting conditions before cardiopulmonary exercise testing. Therefore, the findings should be interpreted as associations between baseline redox-related status and cardiorespiratory fitness, not as evidence that maximal exercise induced changes in ATP, T-AOC, SOD, MDA, or GPX.

Combat sports require repeated bursts of high-intensity effort with limited recovery, so athletes may present similar performance profiles on conventional testing while differing in the physiological mechanisms that support aerobic function and recovery. Against this background, identifying biomarkers that help explain interindividual variability in cardiorespiratory fitness is of practical relevance for training monitoring and athlete management. In this pilot cross-sectional study, combat sport athletes showed overall high aerobic capacity, and the most important finding was that resting total antioxidant capacity emerged as the most consistent correlate of both VO_2_max and relative VO_2_max. This signal was observed not only in simple correlation analysis, but also after adjustment for body size, in multivariable regression, decision tree modeling, and robustness analyses, indicating that the association was stable across analytical approaches rather than driven by a single model. MDA showed a more modest but still potentially meaningful contribution, particularly for relative VO_2_max, whereas ATP, SOD, and GPX did not show independent associations under the present resting cross-sectional conditions. Taken together, these findings help address the current lack of systematic evidence linking resting redox-related biomarkers with cardiorespiratory fitness in combat sport athletes and suggest that integrated antioxidant capacity may have practical value as an informative physiological marker in this population. The observed VO_2_max values were broadly consistent with values reported in previous studies of trained combat sport athletes. However, because the present study did not include a comparison group, it cannot determine whether these athletes had higher cardiorespiratory fitness than the general population or athletes from other sports, reflecting the specific advantage of combat sports in sustaining high-intensity intermittent loads. This aligns with conclusions from prior systematic reviews and cross-sectional studies. Previous work has shown that long-term combat training significantly improves VO_2_max, ventilatory threshold, and heart rate recovery, rendering athletes superior in aerobic capacity compared with the general population (Julio and Franchini, 2021; Wang et al., 2025). While team sports such as soccer and basketball also involve high-intensity exercise, their aerobic fitness is constrained by sport-specific characteristics and tends to be lower than that of combat sport athletes. Intervention studies, such as those by Mathunjwa et al. in taekwondo, further demonstrated that short-term high-intensity interval training (85–95% VO_2_max) can markedly improve VO_2_max and body composition, underscoring the efficiency of combat-specific adaptations (Mathunjwa, 2019). Moreover, differences across combat disciplines have been noted: taekwondo athletes exhibit superior lower-limb power and aerobic capacity, while jiu-jitsu competitors show greater muscular endurance. Building on this, the present study provides adds preliminary evidence linking T-AOC strongly with VO_2_max in combat sport athletes, suggesting that antioxidant status functions not only as a protective factor for physiological homeostasis but also as a biological marker of cardiopulmonary performance.

In comparing oxidative stress and antioxidant defenses, previous research has shown that high-level athletes often exhibit elevated resting lipid peroxidation products alongside remodeling of antioxidant enzyme systems. For instance, Balakrishnan & Anuradha reported higher levels of TBARS and conjugated dienes in athletes, accompanied by increased SOD activity and reduced GPX activity, suggesting that prolonged high-intensity training may disturb the oxidative–antioxidant balance (Balakrishnan and Anuradha, 1998). The absence of significant correlations between SOD, GPX, and VO_2_max in this study diverges from some prior reports, likely due to differences in sample size, measurement timing, or the unique adaptation profiles of combat sport athletes. Although acute and chronic high-intensity exercise may influence oxidative stress, the present study cannot evaluate such exercise-induced responses because no post-exercise blood samples were collected, as evidenced by Devrim-Lanpir et al., who found increased 8-iso-PGF_2_α and total oxidative status in ultra-endurance athletes, with dietary antioxidant intake modulating these effects (Devrim-Lanpir et al., 2020). In the present study, MDA showed a positive correlation with relative VO_2_max, suggesting that although oxidative stress products are not primary determinants, they may contribute to explaining aerobic differences.

It is noteworthy that findings on antioxidant supplementation remain inconsistent. While supplementation with single vitamins such as C or E may lower oxidative stress, they do not significantly improve VO_2_max (Devrim-Lanpir et al., 2020). In contrast, composite supplements or natural compounds such as polyphenols and citrulline have been shown to enhance TAC and mitigate training-induced fatigue to some extent (Hernández-Landa et al., 2024). This study emphasizes that overall antioxidant capacity (T-AOC) more reliably reflects and predicts cardiorespiratory fitness than individual enzymatic markers, indicating that the integrated defense system provides greater explanatory power. These results echo findings from both intervention and observational studies, highlighting the need for further exploration of the relationship between antioxidant defenses and cardiorespiratory fitness in combat sport athletes.

Previous research has suggested a coupling relationship between aerobic capacity and antioxidant levels, with endurance athletes displaying higher VO_2_max, stronger T-AOC, and lower lipid peroxidation compared with non-athletes. General population studies also report positive associations between T-AOC and VO_2_max. However, such evidence has been lacking in combat sport athletes. To our knowledge, this pilot study provides preliminary evidence of a positive association between resting T-AOC and VO_2_max in combat sport athletes, reinforcing that comprehensive antioxidant capacity, rather than isolated enzymatic markers, provides a more stable reflection of differences in cardiorespiratory fitness. By contrast, findings on SOD and GPX have been inconsistent across studies: high-level athletes often show elevated lipid peroxidation at rest, with SOD activity upregulated and GPX activity downregulated, reflecting training-induced remodeling (Bogdanis, 2012). Endurance intervention trials also reveal that changes in SOD and GPX are highly sensitive to training mode and timing (Thirupathi et al., 2020). Such methodological, sample, and nutritional differences may explain discrepancies and highlight the limited reliability of single-enzyme indicators.

Importantly, antioxidant capacity does not automatically translate into performance gains (Mason et al., 2020). For example, supplement-induced increases in T-AOC in taekwondo athletes do not necessarily coincide with improvements in VO_2_max, underscoring that while antioxidant status reflects metabolic and defense capacity, it is not the sole determinant of performance (Xu et al., 2025). The present findings reinforce the central role of overall antioxidant capacity in the cardiorespiratory fitness of combat sport athletes. ROS generated as an unavoidable by-product of skeletal muscle metabolism during exercise, contribute to oxidative stress through elevated lipid peroxidation and protein oxidation during prolonged high-intensity training. Yet, regular training induces adaptive upregulation of antioxidant systems—including SOD, GPX, and catalase (CAT)—facilitating a bidirectional mechanism of “moderate damage–beneficial adaptation (Daniela et al., 2022; Powers et al., 2023).” This phenomenon, repeatedly validated in exercise physiology, explains the observed close association between cardiorespiratory fitness and antioxidant status in combat sport athletes. The strong positive correlation between T-AOC and VO_2_max in this study illustrates the critical role of overall antioxidant potential in maintaining aerobic capacity. As an integrative measure, T-AOC encompasses both enzymatic and non-enzymatic components—including glutathione, uric acid, bilirubin, and various lipid- and water-soluble vitamins—thus offering a comprehensive reflection of systemic reducing capacity. Compared with individual enzymatic indicators such as SOD and GPX, T-AOC is less sensitive to transient testing conditions or short-term load fluctuations and more reliably represents the cumulative influence of long-term training and recovery. This stability accounts for its consistent association with cardiorespiratory fitness in cross-sectional analyses.

Compared with the stability of T-AOC, findings regarding SOD and GPX are often inconsistent. In this study, neither demonstrated significant associations with VO_2_max, which may be attributable to differences in assay methods, sample size, or the specific characteristics of combat sports (Stojiljković et al., 2024). Previous studies have shown that athletes at rest frequently present elevated lipid peroxidation levels, accompanied by increased SOD activity and decreased GPX activity, suggesting a remodeling of the antioxidant system (Özdemir and Demir, 2025). However, these enzymatic markers are highly sensitive to exercise cycles, sampling time points (pre- or post-exercise), and biological matrices (plasma or erythrocytes), making discrepancies between studies difficult to avoid. By contrast, MDA, as a terminal product of lipid peroxidation, is a classical marker of membrane lipid damage (Zheng et al., 2024).The present results revealed a moderate positive correlation between MDA and relative VO_2_max, indicating that MDA may partially reflect differences in aerobic capacity. Nevertheless, MDA levels are also influenced by diet, inflammation, and methodological factors, making its relationship with aerobic indicators context-dependent (Özdemir and Demir, 2025). When antioxidant defenses are sufficient, moderate elevations of MDA may simply reflect recent training load and membrane lipid turnover. Conversely, when recovery is insufficient or antioxidant reserves are depleted, elevated MDA suggests accumulated damage, potentially impairing oxygen transport and utilization (Sayar et al., 2025). Thus, the association between MDA and cardiorespiratory fitness is not fixed but depends on training background and recovery status.

ATP, SOD, and GPX did not show independent associations with cardiorespiratory fitness in the present study. Resting plasma ATP may not adequately reflect exercise-related energy turnover, whereas SOD and GPX are highly sensitive to training load, recovery status, and sampling conditions (Thirupathi et al., 2020; Cardoso et al., 2021; Stojiljković et al., 2024). Together with the small and relatively homogeneous sample (Abt et al., 2020), this may explain the null findings, which should be interpreted cautiously.

Integrating energy metabolism with vascular regulatory mechanisms, a mechanistic chain of “ATP–purinergic signaling–blood flow redistribution” can be established. During exercise, skeletal muscle under hypoxia and shear stress promotes ATP release from erythrocytes and endothelial cells. Accordingly, the lack of an independent ATP signal in the present data does not necessarily contradict its physiological importance during exercise, but rather suggests that resting plasma ATP may be less suitable than integrated antioxidant markers for cross-sectional prediction of cardiorespiratory fitness. Extracellular ATP acts on P2Y_1_ and P2Y_2_ receptors, activating nitric oxide (NO), prostacyclin, and endothelial hyperpolarization pathways, thereby inducing vasodilation and functional hyperemia to enhance muscle perfusion (Sudi et al., 2023). ATP is then hydrolyzed to ADP, AMP, and adenosine, with adenosine acting on A_2_ receptors to further amplify vasodilation, ensuring effective oxygen and substrate delivery within the microcirculation (Zhang et al., 2021). This process is critical for supporting peak VO_2_ and relies on antioxidant defenses to buffer ROS. The strong correlation observed between T-AOC and VO_2_max in this study can thus be understood as a reflection of robust antioxidant capacity reducing ROS-mediated depletion of NO and membrane integrity, thereby preserving purinergic and NO signaling pathways and optimizing oxygen delivery and utilization. Meanwhile, MDA levels may represent either “adaptive load” or “cumulative damage,” which helps explain the inconsistent directions of MDA–aerobic capacity associations reported in prior studies. Collectively, the findings reveal a multilayered chain of antioxidant defense, lipid peroxidation, and energy signaling in shaping cardiorespiratory fitness among combat sport athletes, underscoring T-AOC as the most representative integrative biomarker and highlighting MDA as a supplementary indicator for understanding sport-specific physiological adaptations.

The study demonstrated a stable and significant association between resting T-AOC and cardiorespiratory fitness in combat sport athletes, suggesting that T-AOC may serve as a potential biomarker for understanding aerobic variability in this specific population. Compared with traditional functional testing, plasma T-AOC measurement may provide supplementary information for monitoring combat sport athletes, but its practical utility requires confirmation in larger longitudinal studies. In the present resting cross-sectional design, MDA should be interpreted only as an exploratory redox-related correlate and cannot be used to infer acute exercise-induced oxidative stress, training stress, or recovery status without post-exercise and recovery-phase sampling.

Overall, resting T-AOC was consistently associated with cardiorespiratory fitness in this pilot sample and may warrant further investigation as a supplementary marker of aerobic variability in combat sport athletes. MDA should be regarded as an exploratory correlate. Because the study was cross-sectional and used a single pre-exercise blood sample, neither marker can be used to infer causality, acute exercise responses, training load, recovery status, or talent potential. Larger longitudinal studies with repeated sampling are needed to evaluate their practical utility.

### Limitations

1. The study included only 21 combat sport athletes from a single institute. Although small samples are common in high-performance and combat sport research because recruitment under standardized conditions is inherently constrained (Abt et al., 2020; Kirk et al., 2020; Hecksteden et al., 2022), this still limits statistical power, external validity, and the stable detection of moderate associations. Therefore, the present findings should be interpreted as preliminary associative evidence from a pilot cross-sectional study rather than broadly generalizable conclusions.
2. The cross-sectional design allows only associations to be observed, without establishing causal direction. Future research should employ longitudinal follow-up or interventional designs to validate these findings.
3. Measurements were restricted to energy metabolism and antioxidant systems, without inclusion of inflammatory markers, mitochondrial function, or molecular signaling pathways. Future studies should expand the scope of indicators to construct a more comprehensive physiological framework. Future studies should recruit larger and more diverse samples, ideally across multiple centers, and use longitudinal designs to verify the stability, temporal direction, and generalizability of the observed associations.
4. Biomarkers were measured only once under resting fasting conditions before maximal exercise testing. Therefore, this study cannot evaluate acute exercise-induced oxidative stress, post-exercise antioxidant activation, or recovery-phase biomarker kinetics. Future studies should include repeated blood sampling before exercise, immediately after exercise, and during recovery.

## 5 Conclusions

Resting T-AOC was positively associated with VO_2_max and relative VO_2_max in this pilot sample of combat sport athletes, whereas the MDA finding was exploratory and ATP, SOD, and GPX showed no consistent associations. These cross-sectional, pre-exercise data do not establish causality, training effects, recovery status, or acute exercise responses. Larger longitudinal studies with repeated biomarker measurements are needed to confirm these findings.

## Data Availability

The datasets presented in this study can be found in online repositories. The names of the repository/repositories and accession numbers can be found in the article/supplementary material.
